# Reflection-Boosted Wearable Ring-Type Pulse Oximeters for SpO_2_ Measurement with High Sensitivity and Low Power Consumption

**DOI:** 10.3390/bios13070711

**Published:** 2023-07-05

**Authors:** Min Gyu Joo, Dae Hyeong Lim, Kyu-Kwan Park, Jiwon Baek, Jong Min Choi, Hyoung Won Baac

**Affiliations:** 1 Department of Electrical and Computer Engineering, Sungkyunkwan University, Suwon 16419, Republic of Korea; 2 Department of Digital Media Communication Engineering, Sungkyunkwan University, Suwon 16419, Republic of Korea; 3 Health H/W R&D Group, Samsung Electronics, Suwon 16677, Republic of Korea

**Keywords:** wearable healthcare device, biophotonics, Monte Carlo simulation, ring-type pulse oximeter, oxygen saturation, photoplethysmography

## Abstract

In this study, we demonstrated a Monte Carlo simulation to model a finger structure and to calculate the intensity of photons passing through tissues, in order to determine optimal angular separation between a photodetector (PD) and a light-emitting diode (LED), to detect SpO_2_. Furthermore, our model was used to suggest a mirror-coated ring-type pulse oximeter to improve the sensitivity by up to 80% and improve power consumption by up to 65% compared to the mirror-uncoated structure. A ring-type pulse oximeter (RPO) is widely used to detect photoplethysmography (PPG) signals for SpO_2_ measurement during sleep and health-status monitoring. Device sensitivity and the power consumption of an RPO, which are key performance indicators, vary greatly with the geometrical arrangement of PD and LED within the inner surface of an RPO. We propose a reflection-boosted design of an RPO to achieve both high sensitivity and low power consumption, and determine an optimal configuration of a PD and LED by performing a 3D Monte Carlo simulation and confirming its agreement with experimental measurement. In order to confirm the reflection-boosted performance in terms of signal-to-noise ratio, R ratio, and perfusion index (PI), RPOs were fabricated with and without a highly reflective coating, and then used for SpO_2_ measurement from eight participants. Our simulation allows the numerical calculation of the intensity of photon passing and scattering through finger tissues. The reflection-boosted RPO enables reliable measurement with high sensitivity, resulting in less power consumption for the LED and longer device usage than conventional RPOs without any reflective coating, in order to maintain the same level of SNR and PI. Compared to the non-reflective reference RPO, the reflection-boosted RPO design greatly enhanced both detected light intensity (67% in dc and 322% in ac signals at a wavelength *λ*_1_ = 660 nm, and also 81% and 375% at *λ*_2_ = 940 nm, respectively) and PI (23.3% at *λ*_1_ and 25.5% at *λ*_2_). Thus, the reflection-boosted design not only enhanced measurement reliability but also significantly improved power consumption, i.e., by requiring only 36% and 30% power to drive the LED sources with *λ*_1_ and *λ*_2_, respectively, to produce the device performance of a non-reflective RPO reference. It is expected that our proposed RPO provides long-term monitoring capability with low power consumption and an enhanced PI for SpO_2_ measurement.

## 1. Introduction

Healthcare information optically obtained via continuous human body monitoring is highly useful as the health data can forewarn of disease initiation and development. To facilitate continuous monitoring, wearable optical detection devices have been proposed, showing promising outcomes and capabilities in measuring blood pressure [1], heartbeat [2], oxygen concentration in blood [3], mental stress [4], sleep disorders [5], and body temperature [6]. Despite such potential from these wearable devices, accurate measurement is often hindered by intentional or unintentional body movement and the possible rearrangement of devices [7,8,9,10]. Bulky and heavy devices are more susceptible to this issue. As compact, lightweight, wearable, and low-power optical devices can be a suitable solution, finger-ring-type devices have been recently developed for steady and long-term human body monitoring [11,12]. These require the integration of various components (sensor, battery, micro-controller unit (MCU), and a wireless communication module) into a miniaturized form. However, their device volume is tightly limited, which is more critical than other bulkier devices because a significant portion of volume must be assigned to a battery which supports viable, sensitive, and long-term measurement. This necessitates a ring-type device design for power-efficient measurement and monitoring to achieve both high sensitivity and long-time usage under a limited battery capacity.

Photoplethysmography (PPG) is an optical modality for measuring pulsatile blood flow [13]. Based on PPG, pulse oximeters utilizing a light-emitting diode (LED) and a photodetector (PD) enable the non-invasive and painless measurement of oxygen saturation, measured by pulse oximeter (SpO_2_). SpO_2_ is an important physiological parameter, for example, to recognize apnea and hypopnea caused by the closure of the upper respiratory tract. Pulse oximeters can be divided into two types according to their optical operation, based on either transmittance or reflectance. A transmittance-based pulse oximeter measures light passing through cutaneous tissues, typically at a fingertip or an ear lobe [14], whereas a reflectance type receives light reflected from the tissues, which is widely applied to measure SpO_2_ at various body parts; e.g., wrists, chest, eyes, and finger knuckles [7,15]. The transmittance-based oximeter provides a better signal-to-noise ratio (SNR) (e.g., by ~6 dB) than the reflectance type [7], but it requires a higher operation current for sufficient light emission to transmit photons through the tissues. This suggests that reflectance-type devices would be more favorable for longer-time usage in a wearable medium [8].

Despite such an operational difference, both types of pulse oximeters enable the optical detection of minute changes in a pulsatile blood flow volume within vascular tissue [9]. The amount of SpO_2_ can be monitored by differentiating optical absorption into two wavelengths: e.g., 660 nm (red; *λ*_1_) and 940 nm (near-infrared; NIR; *λ*_2_). Blood volume variation during cardiac cycles (i.e., a higher volume in the systolic phase than in the diastolic phase) generates an oscillatory ac signal in detected light intensity [9]. This determines a perfusion index (PI) for the amount of SpO_2_ defined by the ratio of ac-to-dc signal at each wavelength [16], which varies with the composition and geometrical location of tissues [17], patient’s cardiovascular condition [11,18], and temperature [18].

High PI values are required to reduce the difference between measured SpO_2_ and arterial oxygen saturation (SaO_2_) [19,20]. This can be achieved by designing a device to have an increased photon propagation path through the tissues and then make more optical exposure to blood [12,21]. Previously, an angular separation between PD and LED has been tuned to effectively increase such propagation path [12,22]. However, the elongated photon propagation has raised an issue of increased optical scattering from blood vessels, subcutaneous fat, and dermis within a complex finger structure. This eventually reduces the SNR, due to the reduced amount of detected light, both ac and dc, which is received by PD. It is challenging to maintain high PI values [20,22], together with detecting a large amount of light that can achieve a reliable SNR. Previously, a nominal SNR of 15 dB has been proposed as a minimum condition required to obtain reasonable SpO_2_ [10].

Ray propagation approaches for optical modeling of non-biological structures include optical transmission, reflection, and absorption [11]. However, these are not suitable for biological tissue cases where diffused light scattering occurs significantly in random directions. A Monte Carlo method, which is a statistical approach available for optical simulation, allows the calculation of photon paths randomly scattered, absorbed, and reflected. Although a complex finger structure has been investigated by using this approach, containing scatterers such as red blood cells and various tissue layers [23,24], such modeling and analysis are not reliable yet especially for optical designs of ring-type pulse oximeters (RPOs) [11,21]. This requires numerical investigation in depth considering accuracy, signal sensitivity, and the dependence on the number of photons [25].

We propose a reflection-boosted, power-efficient, and wearable RPO wherein the inner surface of the ring has a highly reflective metallic coating working as an optical mirror. This design is supported by a 3-dimensional (3D) Monte Carlo simulation, fully taking into account diffused light scattering within a finger. Both PI values and R ratio are estimated along with PD locations within the ring, which are designated by angular separation between PD and LED (*θ_PL_*). Then, we determine an optimal separation angle (*θ_opt_*) to achieve a high PI value, maintaining the RPO reliability simultaneously in a balanced manner. The RPOs designed with and without the reflective coating (defined as RPO-R and RPO-NR, respectively) are fabricated and characterized with human fingers (eight participants). The measured results agree with the Monte Carlo simulation in which the reflection-boosted RPO design greatly enhances both detected light intensity and PI as compared to the reference case without the reflective coating. Our reflection-boosted wearable RPO configuration enables the significant improvement of power consumption, providing a long-term monitoring capability with the enhanced PI values for SpO_2_ measurement.

## 2. Materials and Methods

### 2.1. Monte Carlo Simulation for Ring-Type Pulse Oximeters

**Figure 1:**
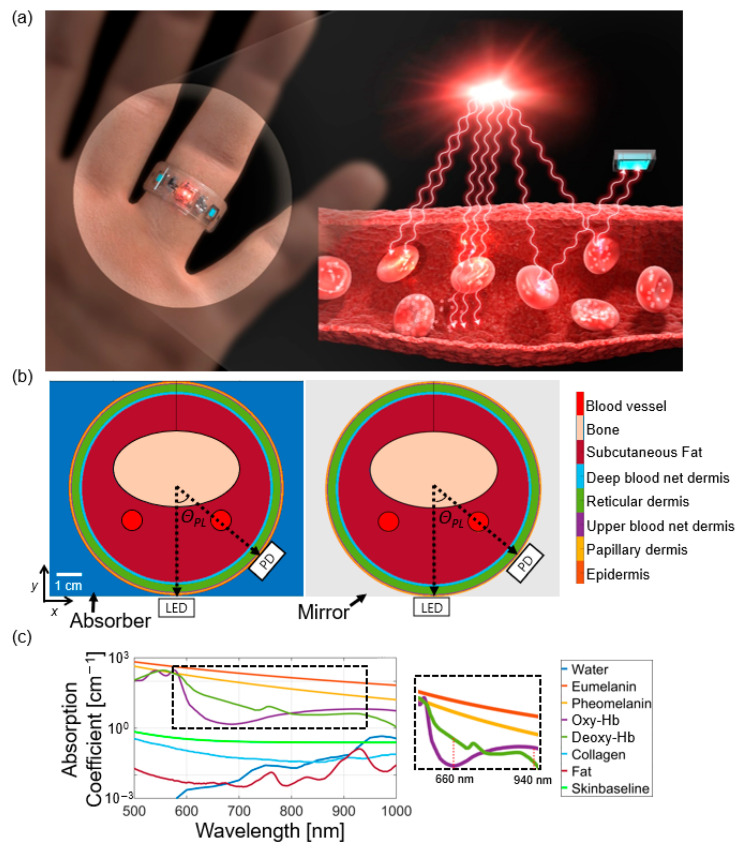
(**a**) Schematic illustration of the finger-type wearable RPO (**left**) and optical measurement process (**right**). The right figure shows optical irradiation of blood vessel via an LED, containing red blood cells, and detection of diffused light via scattering, absorption, and reflection by a PD; (**b**) cross-sectional simulation domains for the finger structure without (**left**) and with the surrounding mirror (**right**). The angular separation between PD and LED is defined by *θ_PL_*. A detailed structural geometry with cross-sectional tissue composition is available in Appendix B; (**c**) absorption spectra of constituent elements within the tissue.

We utilized the Monte Carlo method [23,24,25] (detail described in Appendix A) to calculate photon propagation, including reflection, absorption, and scattering, along with photon paths in human tissues, as illustrated in Figure 1a. The finger structure for simulation was modelled with constituent tissue layers (i.e., epidermis, dermis, subcutaneous fat, bone, and blood vessels) [26,27] (Figure 1b) with the geometrical information provided in Table 1. The dermis was again divided into four different layers (papillary dermis, upper blood net dermis, reticular dermis, and deep blood net dermis) [27]. Two finger arteries were located beneath the finger bone [21]. Veins were not considered due to their random distribution in persons. For photon path calculation, the LEDs with two wavelengths (*λ*_1_ and *λ*_2_) were placed at the bottom of the finger. Then, for angle-dependent calculation, the PD was positioned with *θ_PL_* between 25° and 95° along the rim of the ring. Within this angular range, the calculation was repeated by relocating the PD by an interval of 5° (i.e., 25°, 30°, 35°, etc.). Then, photon propagation was calculated using two structures: one is surrounded by a light absorber for RPO-NR, and the other surrounded by a highly reflective mirror for RPO-R.

An optical absorption coefficient of each tissue layer was calculated by using the volumetric fraction of constituent materials such as blood, water, fat, melanin, skin baseline, and collagen. Their absorption coefficients are shown in Figure 1c [27,28,29] (see Appendix B). Variation of SpO_2_ (i.e., the ratio of deoxyhemoglobin; deoxy-Hb and oxyhemoglobin; oxy-Hb) was set from 70% to 100% with an interval of 10%. During a cardiac cycle, the digital artery radius and the blood volume fraction in capillaries inside the tissues were also set as summarized in Table 2 [30]. The ac signal of the detected light was obtained by subtracting the detected light intensity in the systolic state from that of the diastolic state, the latter of which is the dc intensity.

### 2.2. Determination of Number of Photons for Accurate Simulation

**Figure 2:**
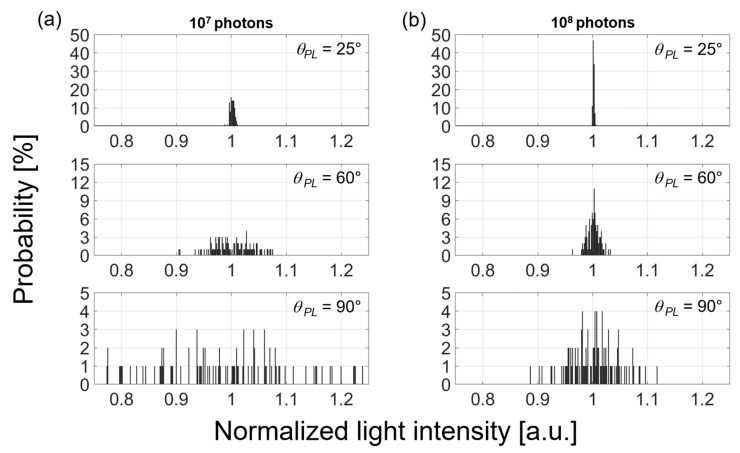
Calculated probability distributions of the detected light intensities along the number of photons (10^7^ and 10^8^) shown in (**a**,**b**), respectively, each of which is displayed with three different *θ_PL_*. A single event of optical transmission from LED and detection at PD was repeated 100 times. An average of detected intensity was set to 1 (i.e., shown as a center of horizontal axis). For example, the top-left figure (10^7^ number of photons and the angular separation of 25°) shows that the average value (1.0) was detected 22 times among 100 events (i.e., 22%).

In order to perform an accurate 3D Monte Carlo simulation, the number of photons should be determined under a given arrangement. We first calculated the detected light intensities at PD for the finger geometry, depending on the angular separation between LED and PD, for light emission from the LED location with various numbers of photons (10^7^, 4 × 10^7^, 7 × 10^7^, and 10^8^) in the finger model shown in Figure 1. Figure 2 shows two examples of photon numbers of 10^7^ and 10^8^, each of which represents three angular cases (each calculated 100 times to obtain the probability). As the number of photons launched from the LED increased, the calculation accuracy was improved. The detected light intensity at the PD was 0.356%, 0.0262%, and 0.0012% at *θ_PL_* = 25°, 60°, and 90°, respectively. Figure 2 also demonstrates that the detected light intensity deviated from its average more frequently as *θ_PL_* increased. In the case of 10^7^ photons, the standard deviations of 0.0039, 0.0356, and 0.1278 at *θ_PL_* = 25°, 60°, and 90° were obtained. This means that 99.73% of the photons are concentrated within only ±1.17% from the average intensity at *θ_PL_* = 25°. Then, the photons spread over ±10.68% from the average at *θ_PL_* = 60° and greatly over ±38.34% at *θ_PL_* = 90°, respectively. However, such deviation for the 10^8^ photons in Figure 2b was significantly improved with the standard deviations of 0.0014, 0.0112, and 0.0425, each of which shows 99.73% of the photons falling within ±0.42% at *θ_PL_* = 25°, ±3.36% at *θ_PL_* = 60°, and ±12.75% at *θ_PL_* = 90° from each average intensity.

The photon number dependence shown above agrees with results previously reported elsewhere. Previous simulations for the tissues [25] reported that predictable and reliable results from a reference curve could be obtained with 10^8^ photons, whereas the cases of 10^6^ and 10^7^ photons showed deviation. Established studies on RPOs have also reported that 10^7^ photons launched from an LED is not sufficient to calculate a reasonable SpO_2_ [12,21]. Our calculation suggests that reliable SpO_2_ measurement requires a minimum number of photons emitted from the LED.

### 2.3. Experimental Conditions for Ring-Type Pulse Oximeters

**Figure 3:**
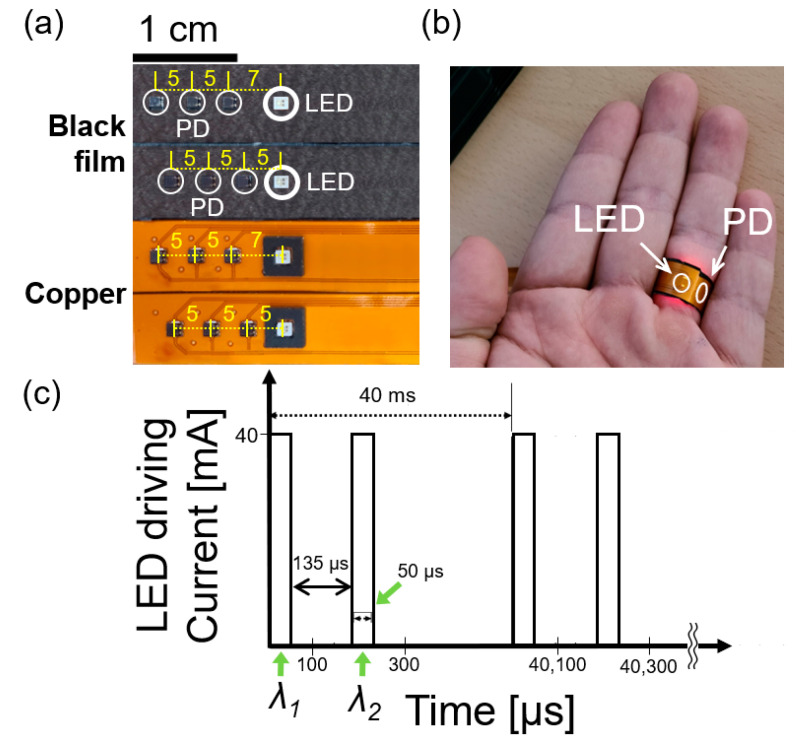
(**a**) Fabricated RPOs including LED and PDs mounted on FPCBs (1.1 cm width and 7.2 cm length). Both of non-reflective and reflective RPOs (RPO-NR and RPO-R) have a common arrangement of LED and PDs while the RPO-NR is coated with a black absorbing film on the FPCB substrate (reflectance < 10%; top image), and the RPO-R is coated with a 25 mm thick copper (reflection > 90%). Six different PD positions were chosen and mounted on FPCBs (5, 7, 10, 12, 15, and 17 mm from the LED). One set has PDs 5, 10, and 15 mm apart from the LED, and the other with PDs 7, 12, and 17 mm apart from the LED. One end of the FPCB was connected to a main control board to operate LED and PDs; (**b**) an example image with the ring-type RPO encircling a finger; (**c**) a timing diagram for LED operation. Each light source was driven with a duty cycle of 0.125%. For operation wavelengths, *λ*_1_ = 660 nm and *λ*_2_ = 940 nm, respectively.

We experimentally obtained PPG signals from eight participants using RPO-NR and RPO-R. All participants has the skin tone typical in Asia. RPO-NR has a black absorber as shown in the top of Figure 3a (reflectance < 10%), while RPO has a highly reflective copper coating (reflectance > 90%) as shown in the bottom of Figure 3a [31]. The FPCB was mounted with a LED package (SFH 7016; OSRAM; Munich, Germany) located at the center (1.85 mm × 1.65 mm in dimensions), and three PDs (2 mm × 1.8 mm in dimensions; SFH 2704; OSRAM; Munich, Germany) with different positions from the LED. Two sets were prepared for PD measurement at distances of 5, 7, 10, 12, 15, and 17 mm from the LED location. Each different PD position can be represented by an angle between PD and LED as *θ_PL_*; *θ*_1_, *θ*_2_, *θ*_3_, *θ*_4_, *θ*_5_, and *θ*_6_, respectively. We noted that the angular locations (*θ*_1_~*θ*_6_) varied slightly with the finger diameters of participants. Figure 3b shows an example photograph for RPO measurement. The LED was driven electrically with 40-mA current. As shown in Figure 3c, one period (40 ms) included two 50-μs pulses (on time) and 135-μs interval (off time) between two pulses, which was repeated with a 25-Hz rate. Two pulses made sequential emission of pulsed optical outputs with two wavelengths of *λ*_1_ and *λ*_2_.

## 3. Results and Discussion

**Figure 4:**
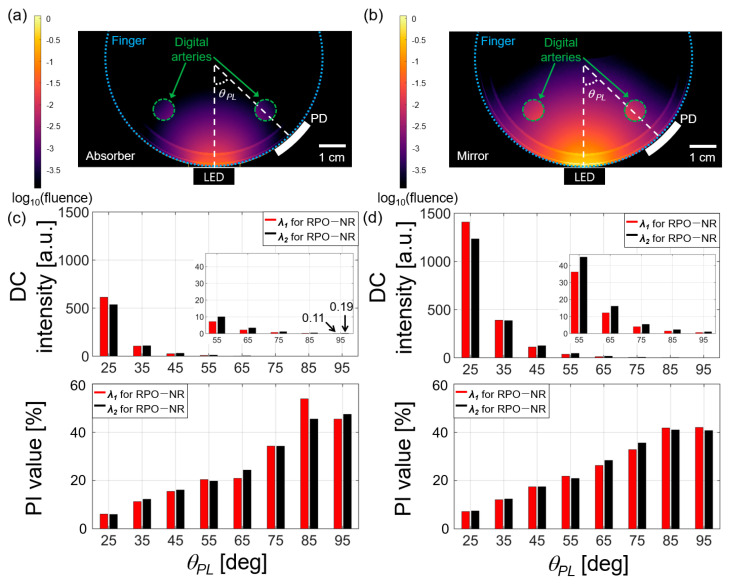
Calculated light intensities and PI values for RPOs; (**a**,**b**) 2D cross-sectional profiles of dc light intensity distributions (left: RPO-NR; right: RPO-R) for the human finger model with two artery vessels (indicated with the dotted white circles). LEDs and PDs are shown in contact with the finger tissue surface; (**c**,**d**) the dc intensity and the PI value along with the PD location in terms of angular separation from the LED, *θ_PL_* = 25°~95° (here, *λ*_1_ = 660 nm and *λ*_2_ = 940 nm). These dc intensity and PI values were obtained by taking an integration over the PD area. The insets for the dc intensity figures show enlarged views for the range of 55°~95°.

Then, we performed 3D simulations for RPO-NR and RPO-R configurations in order to find the dc light intensity and the PI value (Figure 4). Cross-sectional views for the dc intensity distributions are shown in Figure 4a,b. In the simulation, we assumed a fully absorbing medium that surrounds the finger in RPO-NR (i.e., 100% absorption), and a fully reflective surrounding medium in RPO-R (i.e., 100% reflectance). The comparison of two dc intensity distributions in Figure 4a,b confirms that RPO-R greatly enhances the detected light intensity via PD, which is boosted by strong reflection at the finger–mirror boundary. For example, when the dc intensities at *θ_PL_* = 25° are compared for both cases of RPO-NR (Figure 4c) and RPO-R (Figure 4d), RPO-R exhibits relatively enhanced intensities Δ*I_dc_* which are 129.7% for *λ*_1_ = 660 nm and 130.3% for *λ*_2_ = 940 nm, respectively. Although the intensity drops rapidly with *θ_PL_* due to photon absorption and scattering in the tissues, a several-fold enhancement is observed over the entire angular range.

**Figure 5:**
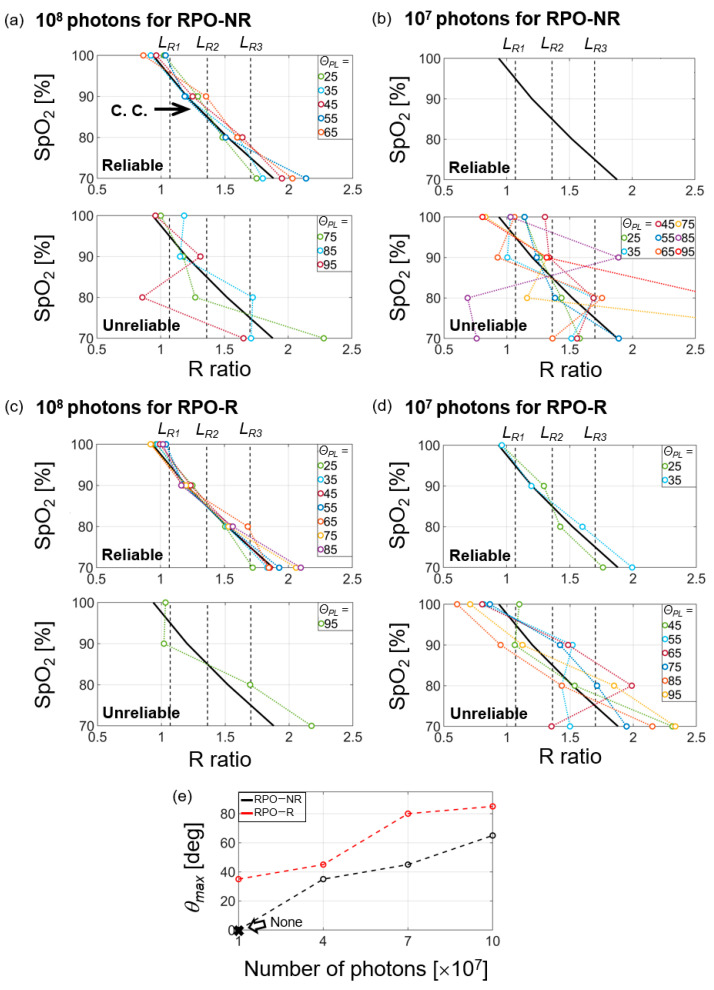
SpO_2_ versus R ratio (calculation) shown with reliability limits in (**a**–**d**), i.e., denoted as vertical dotted lines determined from the calibration curve (C. C.). All the calculation was performed assuming four different SpO_2_ measurements (70, 80, 90, and 100%): (**a**) RPO-NR with 10^8^ photons; (**b**) RPO-NR with 10^7^ photons; (**c**) RPO-R with 10^8^ photons; (**d**) RPO-R with 10^7^ photons; (**e**) the maximum angular separation of *θ_PL_* (=*θ_max_*) versus the given number of photons for RPO-NR (black dotted line) and RPO-R (red dotted line). The R ratio was obtained for a fixed angular location of PD (*θ_PL_*) (i.e., each single curve consists of four data points obtained along with the SpO_2_). The R ratio calculation was repeated over *θ_PL_* = 25°~95°. A calibration curve for SpO_2_ (the thick, black line marked as C. C.) is common for all figures from (**a**–**d**).

As shown in the bottom of Figure 4c,d, PI gradually increased with *θ_PL_* due to elongated photon paths and then photon exposure to blood [12,21]. Moreover, the PI values of RPO-R for *θ_PL_* < 65° were 10–20% higher than those of RPO-NR because an average photon path length in RPO-R was increased by multiple reflection at the tissue/mirror interface. For *θ_PL_* > 65°, higher PI values for RPO-NR are shown, compared to the RPO-R cases, but this is due to unreliability due to the high deviation of detected light (see Figure 5a).

Our calculated results shown in Figure 4d exhibit the PI values reaching 40–50% which are higher than those previously reported elsewhere (e.g., ~4%) [12]. While a PPG sensor detects blood variation passing through arteries, capillaries, and veins over deep tissue ranges [9], this essentially generates a spatiotemporal signal resulting from the spatial movement of blood and the temporal variation during cardiac cycles. In our simulation, we simultaneously changed the volume fraction of blood involved in whole tissues including arteries, capillaries, and veins, which leads to spatially integrated blood signal and thus, a higher peak amplitude in ac signal.

A calibration curve was previously obtained to determine SpO_2_ corresponding to the R ratio [8,9,12]. Based on our simulation results, we obtained the calibration curve to find an optimal angle (*θ_PL_* = *θ_opt_*) giving the maximum PI value over *θ_PL_* together with guaranteeing measurement reliability. To determine the calibration curve (represented as C. C. in Figure 5a–d), at first, we calculated R ratios corresponding to 15 different *θ_PL_* (from 25° to 95°) by varying SpO_2_ (70%, 80%, 90%, and 100%) in the tissue and the number of photons (10^7^, 4 × 10^7^, 7 × 10^7^, and 10^8^) emitted from RPOs. Then, the calibration curve for RPOs was obtained by taking the average of 15 R ratios at each SpO_2_, which were calculated by using the case of 10^8^ photons in RPO-NR (shown in Figure 5a). In order to determine the reliability, three *L_R_* values were defined (vertical dotted lines in Figure 5a–d) as the limit of reliable R values. Each *L_R_* means the median of two R ratios corresponding to each SpO_2_ on the calibration curve: *L_R_*_1_ = 1.075 = $\frac{R_{100\%}+R_{90\%}}{2}=\frac{0.9+1.25}{2}$, *L_R_*_2_ = 1.375 = $\frac{R_{90\%}+R_{80\%}}{2}=\frac{1.25+1.5}{2}$, and *L_R_*_3_ = 1.7 = $\frac{R_{80\%}+R_{70\%}}{2}=\frac{1.5+1.9}{2}$. Thus, for example, *L_R_*_1_ corresponds to the boundary between SpO_2_ values of 100% and 90%. Similarly, *L_R_*_2_ corresponds to the boundary between 90% and 80%; also, *L_R_*_3_ between 80% and 70%. Reliable and unreliable cases along *θ_PL_* are shown at the top and the bottom of each figure, respectively.

The top of Figure 5a shows the relation between R ratio and SpO_2_ in the case of 10^8^ photons for RPO-NR where 25° ≤ *θ_PL_* ≤ 65°. This confirms that all the R ratio values are within the reliable ranges for SpO_2_ = 70, 80, 90, and 100% (i.e., R ratio < *L_R_*_1_, *L_R_*_1_ < R ratio < *L_R_*_2_, *L_R_*_2_ < R ratio < *L_R_*_3_, and R ratio > *L_R_*_3_, respectively). However, for *θ_PL_* > 65° (the bottom of Figure 5a), one or more R ratio values are not in the reliable range. Thus, we define the maximum value of *θ_PL_* as *θ_max_* in which R ratios for each SpO_2_ are maintained within the reliable range. Such *θ_max_* is also equal to *θ_opt_* because PI gradually increases with *θ_PL_* and becomes the maximum at *θ_max_*, within the reliable range. Similarly, Figure 5b–d also show the other cases of photon numbers for RPO-NR and RPO-R. We have confirmed that *θ_max_* increased with the photon numbers launched from LED: *θ_max_* = 35°, 45°, and 65° for photon number = 4 × 10^7^, 7 × 10^7^, and 10^8^, respectively. This is because of the reduced deviation of the detected light intensity with the increased photon number. However, for the case of 10^7^ photons, none of *θ_PL_* values (25°~95°) were within the reliable R ratio (Figure 5b), as with results previously reported elsewhere [17,18].

In comparison between RPO-R and RPO-NR, RPO-R exhibited better performance. With regard to 10^8^ photons, Figure 5c shows that *θ_max_* = 85° in RPO-R, which is 20° higher than that of RPO-NR (65°). This is due to RPO-R with the stronger light intensity reaching PDs, compared to RPO-NR. Furthermore, it should be noted that RPO-R showed *θ_max_* = 35° even for 10^7^ photons (Figure 5d), which was not observed in RPO-NR (i.e., was not measurable). Such enhancement of *θ_max_* with RPO-R was also confirmed in the other numbers of photons; *θ_max_* = 45° for 4 × 10^7^ photons and *θ_max_* = 80° for 7 × 10^7^ photons, whereas they were 35° and 45°, respectively, in the RPO-NR cases (Figure 5e). Remarkably, this improvement of *θ_max_* allowed higher PI under the same initial photon intensity. As an example, in the case of 10^8^ photons, the PI value for *θ_max,__RPO-R_* = 85° was 0.42 at *λ*_1_ = 660 nm, while it was only 0.21 for *θ_max,__RPO-NR_* = 65°, resulting in a 100% enhancement. Similarly, the PI values at *λ*_2_ = 940 nm were 0.41 and 0.24, respectively, exhibiting a 70.8% enhancement as well.

Previously, a reliable range of R ratio when SpO_2_ > 95% has been reported as 0.5–0.7 [21,22,32,33]. Our simulation shows that the R ratio of 0.9 for SpO_2_ = 100%. This difference was possibly caused by slight variation in material properties such as scattering and absorption coefficients used in the simulation.

**Figure 6:**
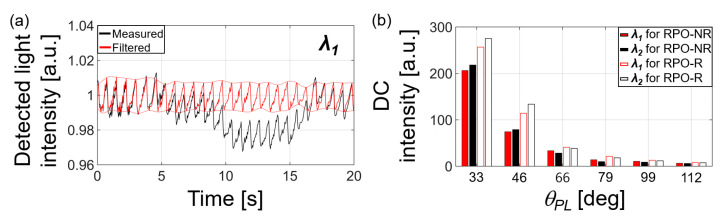
Experimentally measured light intensities (an example from one participant) when SpO_2_ > 95%: (**a**) PPG signals for RPO-R represented in a measured raw form (black) and its filtered form (red; a bandpass filter applied over a frequency range of 0.7–4 Hz). These were obtained with *λ*_1_ = 660 nm and *θ_PL_* = *θ*_1_ (i.e., 33°); (**b**) Intensities of dc signals obtained at different *θ_PL_* (set by six PD locations along the ring).

The experimentally obtained results show that the RPO-R structure significantly improved the detected light intensities. We obtained PPG signals for two wavelengths (*λ*_1_ and *λ*_2_) from eight participants with an SpO_2_ higher than 95%. The measured waveform in Figure 6a shows some distortion due to the dc bias, which is randomly caused by a participant’s movement. Such distortion and system noise were removed (the red line in Figure 6a) by utilizing a bandpass filter over a frequency range from 0.7 to 4 Hz which corresponds to that of the human cardiac cycle. Then, we determined the dc intensity by averaging the local minimum peaks of filtered signal (i.e., the bottom envelope) in Figure 6a. The ac intensities were calculated by differentiating the dc intensity from the average of local maximum peaks of filtered signal (i.e., the top envelope) in Figure 6a. The dc (shown in Figure 6b) and ac intensities over all PD positions decreased with increasing *θ_PL_* from *θ*_1_ to *θ*_6_, each of which corresponds to 33°, 46°, 66°, 79°, 99°, and 112°. The measured dc light intensities for the RPO-R structure were significantly increased as compared to the RPO-NR case, coinciding with the simulation results. For example, the enhancement in dc and ac intensities at *θ*_1_ was 67 and 322 % for *λ*_1_ = 660 nm, and 81 and 375 % for *λ*_2_ = 940 nm, respectively.

**Figure 7:**
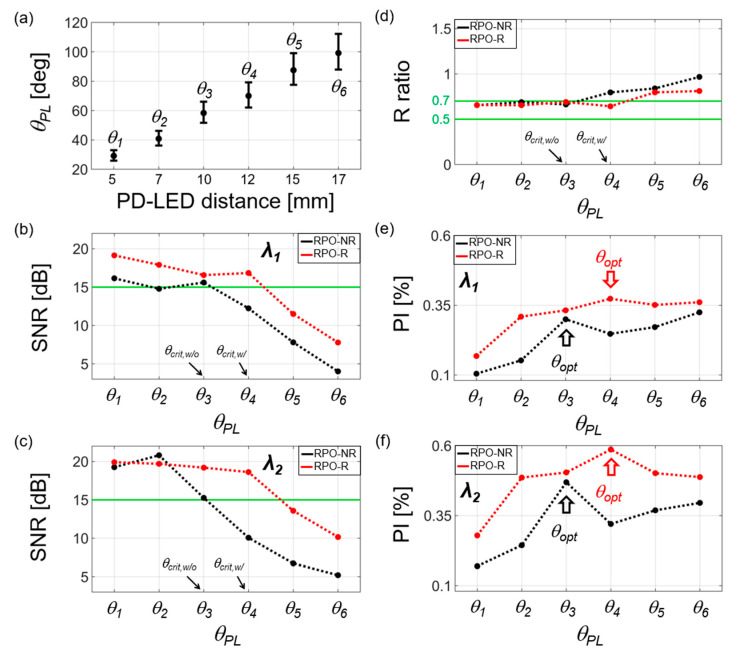
Experimentally measured results of *θ_PL_*, SNR, R ratio, and PI from eight participants with SpO_2_ > 95%: (**a**) *θ_PL_* versus the distance between PD and LED. *θ_PL_* slightly varies with the finger diameter of each participant. The average of *θ_PL_* is denoted by a black dot; (**b**,**c**) SNR values at two wavelengths, *λ*_1_ and *λ*_2_. The reliability limit of SNR is denoted by a green line (set to 15 dB [10]); (**d**) R ratios. The reliable range is denoted by green lines (i.e., 0.5 < R < 0.7 [21,22,32,33]); (**e**,**f**) PI for two wavelengths, *λ*_1_ and *λ*_2_. The optimal angle *θ_PL_* = *θ_opt_* is determined (arrows), satisfying two conditions of the maximum PI and SNR > 15 dB. For all figures, each data point shown as dots (black or red) represents the average value from eight participants.

Figure 7 shows measurement results for *θ_PL_*, SNR, R ratio, and PI from eight participants. For position-dependent characterization of PD from LED within RPO-R and RPO-NR, we compared six different PD locations 5, 7, 10, 12, 15, and 17 mm placed apart from LED. Although such PD-LED lengths were initially fixed by fabrication, the angular location of PD from LED (*θ_PL_*) slightly varied along with participants. Figure 7a shows all PD positions represented in terms of *θ_PL_* = *θ*_1_, *θ*_2_, *θ*_3_, *θ*_4_, *θ*_5_, and *θ*_6_, each of which corresponds to an individual separation length between PD and LED. The variation of *θ_PL_* is due to the different diameter of each participant finger. In Figure 7a, each average values of *θ_PL_* for all participants were denoted as black dots. All other dotted data (red or black) in Figure 7b–e also each denote the average values for participants.

In Figure 7b,c, SNR was obtained by dividing a signal spectrum with the frequency range over 0.7–4 Hz by a noise spectrum over 7–10 Hz. This shows that SNR gradually decreases as *θ_PL_* increases due to the reduction in the detected light intensity. Nevertheless, RPO-R exhibits a relatively higher SNR (red dotted lines in Figure 7b,c) as much as ~4.6 dB at *λ*_1_ = 660 nm and ~8.6 dB at *λ*_2_ = 940 nm, respectively, when compared to the RPO-NR cases (black dotted lines). For reliable RPO measurement, the nominal SNR limit was set to 15 dB [10], represented by green lines in Figure 7b,c. Within this limit (>15 dB), the maximum *θ_PL_* was obtained as *θ*_3_ for RPO-NR and *θ*_4_ for RPO-R, respectively: i.e., each maximum defined as *θ*_3_ = *θ_crit,w/o_* and *θ*_4_ = *θ_crit,w/_*.

Figure 7d shows the result for R ratio. As the reliability of R ratios were previously set as 0.5–0.7 [21,22,32,33] for SpO_2_ > 95%, we utilized such range shown as green solid lines in Figure 7d. In our measurement, this range was satisfied when *θ_PL_* ≤ *θ_crit,w/o_* for RPO-NR and *θ_PL_* ≤ *θ_crit,w/_* for RPO-R. This means that the common reliable range of SNR and R ratios are obtained when *θ_PL_* ≤ 60° for RPO-NR and *θ_PL_* ≤ 70° for RPO-R, respectively.

Then, we set an optimal angle *θ_PL_ = θ_opt_* when the highest PI is obtained among *θ_PL_* within the reliable SNR. In Figure 7e,f, each dot denotes an average of all participants’ PI values for a given *θ_PL_* (top: *λ*_1_ = 660 nm; bottom: *λ*_2_ = 940 nm). This confirms that higher *θ_opt_* is obtained with RPO-R (red arrows; *θ_PL_* ≤ *θ_crit,w/_*) compared to the RPO-NR cases (black arrows; *θ_PL_* ≤ *θ_crit,w/o_*) for both wavelengths. Figure 7e,f also shows that the PI values do not significantly increase at the range of *θ_PL_* > *θ_opt_* while the gradual increase is observed in the simulation results. We note that a constant dc signal could be caused by a minimal gap between finger and FPCB, defined here as a gap-induced dc (dc_gap_), commonly appearing in both RPO-NR and RPO-R [34]. This means that the measured dc intensity is derived from blood in the tissue and dc_tissue_, as well as the dc_gap_. The proportion of dc_gap_ out of the measured signal (i.e., the sum of noise and PPG signal) increased with *θ_PL_*. This is because RPO-R has higher reflection (90%) than RPO-NR (10%). Therefore, the PI values in RPO-R were increased by 23.3% ($=\frac{{PI}_{w/,4}-{PI}_{w/o,3}}{{PI}_{w/o,\;3}}=\frac{0.0037-0.0030}{0.0030}$) at *λ*_1_ = 660 nm and 25.5% ($=\frac{0.0059-0.0047}{0.0047}$) at *λ*_2_ = 940 nm, respectively. Such higher PI in RPO-R, compared to the RPO-NR case, results from the improvement of the ac intensity, which is higher than that of the dc intensity.

Our results demonstrate that RPO-R allows lower power consumption than RPO-NR, because a lower driving current to LED is required in RPO-R to achieve the same light intensity emitted to a finger compared to RPO-NR. For example, the same emission level from LED (SFH 7013; OSRAM, Munich, Germany) [35] was obtained from an only 12-mA current in RPO-R with the condition of *θ_PL_* = 60° and *λ*_1_ = 660 nm (similarly, 11 mA for *λ*_2_ = 940 nm), while RPO-NR consumed 20-mA current for both wavelengths. Such a reduced driving current led to low power consumption, only requiring 36% for *λ*_1_ and 30% for *λ*_2_ to maintain the same performance with RPO-NR. With an increasing demand for low power consumption for wearable devices, various approaches have been previously suggested to reduce the power consumption of pulse oximetry. By reducing the amplitude of LED driving current [36,37] or its duty cycle [38,39], power consumption was reduced by >0.4 mW. However, those approaches also reduced SNR as much as 9.29 dB, often making continuous monitoring difficult. Furthermore, such a low LED intensity decreases PI because the number of photons exposed to blood in the tissue is reduced. However, we have confirmed that the enhancement of PI and low power consumption are simultaneously achieved by the RPO-R structure.

## 4. Conclusions

We have demonstrated the reflection-boosted wearable RPO configuration which enables high sensitivity and low power consumption. This was numerically and experimentally confirmed by comparing two RPO configurations: RPO-R and RPO-NR. In order to check the validity of our numerical modeling, the 3-D Monte Carlo simulation was performed confirming the reduction of detected light intensity when *θ_PL_* increased, which was in line with the experimental measurement. The simulation results show that a certain level of LED intensity corresponding to at least 10^8^ photons is necessary to guarantee measurement reliability and PI values. Such a simulation approach was used to predict the performances of RPO-R and RPO-NR. Then, these were compared with the experimental measurement of SNR, R ratio, and PI from eight participants, all of which were used to determine an optimal PD position from the LED for a reliable but simultaneously low-power measurement. As compared to RPO-NR, RPO-R enabled the enhancement of detected light intensity (67% in dc and 322% in ac signals at *λ*_1_ = 660 nm, and also 81% and 375% at *λ*_2_ = 940 nm, respectively) and PI (23.3% at *λ*_1_ and 25.5% at *λ*_2_). Furthermore, RPO-R could also produce the oximeter performance of RPO-NR by consuming a low driving power for LED, i.e., only 36% and 30% at *λ*_1_ and *λ*_2_, respectively. Our RPO-R with the highly reflective coating at the finger–tissue interface allows reliable and high-sensitivity measurement, which results in less power consumption for the LED and longer device usage than conventional RPOs without the reflective coating. We expect that various optical components, for example, micro- or miniature optical lenses, can be utilized to further optimize the sensitivity and power consumption of RPO-R. Our approaches for modeling and device development would also be useful to predict and improve performance of various wearable optical devices.

## Figures and Tables

**Table 1:** Geometrical information of the finger structure.

Geometrical Information	The Center Location of Tissues											
Value	Bone (x) [cm]			Bone (y) [cm]			Vessel (x) [cm]			Vessel (y) [cm]		
	0			0.3			0.4			0.45		
**Geometrical information**	**Size of geometries**											
Value	Bone (x) [cm]		Bone (y) [cm]		Vessel radius (x) [cm]		Ring diameter [cm]		PD surface [cm^2^]		LED radius [cm]	
	0.5		0.3		0.08		1.7		0.3 × 0.3		0.15	

**Table 2:** Simulation parameters changed during a cardiac cycle.

Parameter	Systolic State	Diastolic State
Artery radius	0.08 cm	0.092 cm
Blood volume fraction in capillaries (normalized to the systolic state)	1	1.5

## Data Availability

The study did not report any data.

## Appendix A. Monte Carlo Simulation

For the simulation, we used MCmatlab [40], an open-source tool for biophotonics, whose operation is based on the Monte Carlo method. Diffused photon scattering was realized by using a reduced scattering coefficient (Equation (A1)) and a phase function (*P_HG_*) via the Henyey–Greenstein scattering model (Equation (A2)) including an anisotropy factor (*g*) in the tissues [23,24,28]:

(A1) $$\mu^{\prime }\left(\lambda \right)=S_{a}(f_{Ray}{\left(\frac{\lambda }{\lambda_{0}}\right)}^{-4}+(1-f_{Ray}){\left(\frac{\lambda }{\lambda_{0}}\right)}^{-S_{b}}){\left[\mathrm{cm}\right]}^{-1}$$

(A2) $$P_{HG}\left(\theta \right)=\frac{1}{4\pi }\frac{1-g^{2}}{{\left(1+g^{2}-2gcos\theta \right)}^{3/2}}$$

where $\int_{0}^{\pi }P_{HG}(\theta )sin\theta d\theta =\frac{1}{2\pi }$ and $\int_{0}^{\pi }P_{HG}\left(\theta \right)cos\theta sin\theta d\theta =\frac{g}{2\pi }$. Here, *μ^′^* and *S_a_* denote the reduced scattering coefficient at the specific wavelength (*λ*) and a reference wavelength (*λ*_0_), respectively. *f_Ray_* is the proportion of Rayleigh scattering in the entirety of the scattering of photons. *S_b_* represents a variable dependent on the size of particles in the tissue (values between 0.37 and 4) in which the particle size is larger than 1 μm for Mie scattering and 0.01 μm for Rayleigh scattering, respectively [41]. Values for those parameters are listed in Table A1.

**Table A1:** Specific parameters used for reduced scattering coefficients.

Tissue Layers	*S_a_*	*S_b_*	*f_Ray_*	λ_0_	*g*
Epidermis	66.7	0.69	0.29	500	0.92
Dermis	43.6	0.56	0.41	500	0.92
Blood	825	1.23	0	700	0.98
Bone	8.37	0.64	0	600	0.93
Subcutaneous fat	19.3	0.45	0.17	500	0.95

## Appendix B. Absorption Coefficient of Tissues

The absorption coefficient of each tissue is given by

(A3) $$\mu_{em}\left(\lambda \right)=6.6\times 10^{11}\times \lambda^{-3.33}{\left[\mathrm{cm}\right]}^{-1}$$

(A4) $$\mu_{pm}\left(\lambda \right)=2.9\times 10^{15}\times \lambda^{-4.75}{\left[\mathrm{cm}\right]}^{-1}$$

(A5) $$\mu_{base}\left(\lambda \right)=0.244+85.3\times e^{-(\lambda -154)/66.2}{\left[\mathrm{cm}\right]}^{-1}$$

(A6) $$\mu_{epi}\left(\lambda \right)=C_{m}\left(\beta_{m}\mu_{em}\left(\lambda \right)+\left(1-\beta_{m}\right)\mu_{pm}\left(\lambda \right)\right)+(1-C_{m})\mu_{base}{\left[\mathrm{cm}\right]}^{-1}$$

(A7) $$\mu_{derm}\left(\lambda \right)=C_{h}\left(\gamma \mu_{oxy}\left(\lambda \right)+\left(1-\gamma \right)\mu_{deoxy}\left(\lambda \right)\right)+(1-C_{h})\mu_{base}{\left[\mathrm{cm}\right]}^{-1}$$

(A8) $$\mu_{tissue}\left(\lambda \right)=(B\left(S\mu_{oxy}\left(\lambda \right)+\left(1-S\right)\mu_{deoxy}\left(\lambda \right)\right)+W\mu_{w}\left(\lambda \right)+F\mu_{f}\left(\lambda \right)\;+M\left(0.5\times \mu_{em}\left(\lambda \right)+0.5\times \mu_{pm}\left(\lambda \right)\right)+C\mu_{col}\left(\lambda \right))/(B+W+F+M+C){\left[\mathrm{cm}\right]}^{-1}$$

where *μ_em_*, *μ_pm_*, *μ_base_*, *μ_epi_, μ_derm_*, *μ_tissue_*, *μ_oxy_*, *μ_deoxy_*, *μ_w_*, *μ_f_*, and *μ_col_* are absorption coefficients of eumelanin, pheomelanin, skin baseline, epidermis, dermis, specific tissue component (e.g., blood, bone, subcutaneous fat), oxy-Hb, deoxy-Hb, water, fat, and collagen at specific wavelengths, respectively. *C_m_*, *β_m_*, *C_h_*, and γ are the volume fraction of melanin in the epidermis, the ratio of eumelanin to pheomelanin, the hemoglobin fraction in dermis and epidermis, and the blood oxygenation ratio of deoxy- to oxy-Hb, respectively. Values of those parameters are summarized in Table A2 and Table A3. The optical properties of the epidermis and dermis were derived using Equations (A6) and (A7) [29].

**Table A2:** Specific parameters used for calculation of absorption coefficients of dermis. *C_h,sys_* (*C_m,sys_*) and *C_h,dia_* (*C_m,dia_*) denote the hemoglobin (melanin) fraction in systolic and diastolic states, respectively.

Dermis	*C_h,sys_* (*C_m,sys_* in Epidermis)	*C_h,dia_* (*C_m,dia_* in Epidermis)	*γ* (*β_m_* in Epidermis)
Epidermis	0.001	0.001	0.5
Papillary dermis	0.05	0.076	0.75 × SpO_2_
Upper blood net dermis	0.2	0.304	0.75 × SpO_2_
Reticular dermis	0.04	0.061	0.75 × SpO_2_
Deep blood net dermis	0.1	0.152	0.75 × SpO_2_

**Table A3:** Specific parameters used for absorption coefficients of tissue layers. *B_sys_* and *B_dia_* denote the blood volume fraction at systolic and diastolic states, respectively.

Tissue Layers	*B_sys_*	*B_dia_*	*S*	*W*	*F*	*M*	*C*
Blood	1	1	SpO_2_	0.21	0	0	0
Bone	0.02	0.02	0.87 × SpO_2_	0.31	0.8	0	0.041
Subcutaneous fat	0.07	0.11	0.82 × SpO_2_	0.35	0.65	0.65	0

## Appendix C. SpO_2_ Calculation

The calibration curve of SpO_2_ is obtained by calculating the R ratio which is defined by the ratio of PI values at two wavelengths *λ*_1_ and *λ*_2_ [23,24]:

(A9) $$R=\frac{{ac}_{\lambda_{1}}/{dc}_{\lambda_{1}}}{{ac}_{\lambda_{2}}/{dc}_{\lambda_{2}}}$$

where *dc* and *ac* signals are defined as the light intensity of the diastolic state and the difference of light intensities between the systolic and diastolic states in cardiac cycles, respectively.
